# Fructose stimulates GLP-1 but not GIP secretion in mice, rats, and humans

**DOI:** 10.1152/ajpgi.00372.2013

**Published:** 2014-02-13

**Authors:** Rune E. Kuhre, Fiona M. Gribble, Bolette Hartmann, Frank Reimann, Johanne A. Windeløv, Jens F. Rehfeld, Jens J. Holst

**Affiliations:** ^1^Department of Biomedical Sciences, NNF Centre for Basic Metabolic Research, the Panum Institute, University of Copenhagen, Copenhagen, Denmark;; ^2^Cambridge Institute for Medical Research and MRC Metabolic Diseases Unit, University of Cambridge, Cambridge, United Kingdom; and; ^3^Department of Clinical Biochemistry, Rigshospitalet, University of Copenhagen, Copenhagen, Denmark

**Keywords:** enteroendocrine axis, gastric inhibitory peptide, glucagon-like peptide-1

## Abstract

Nutrients often stimulate gut hormone secretion, but the effects of fructose are incompletely understood. We studied the effects of fructose on a number of gut hormones with particular focus on glucagon-like peptide 1 (GLP-1) and glucose-dependent insulinotropic polypeptide (GIP). In healthy humans, fructose intake caused a rise in blood glucose and plasma insulin and GLP-1, albeit to a lower degree than isocaloric glucose. Cholecystokinin secretion was stimulated similarly by both carbohydrates, but neither peptide YY_3–36_ nor glucagon secretion was affected by either treatment. Remarkably, while glucose potently stimulated GIP release, fructose was without effect. Similar patterns were found in the mouse and rat, with both fructose and glucose stimulating GLP-1 secretion, whereas only glucose caused GIP secretion. In GLUTag cells, a murine cell line used as model for L cells, fructose was metabolized and stimulated GLP-1 secretion dose-dependently (EC_50_ = 0.155 mM) by ATP-sensitive potassium channel closure and cell depolarization. Because fructose elicits GLP-1 secretion without simultaneous release of glucagonotropic GIP, the pathways underlying fructose-stimulated GLP-1 release might be useful targets for type 2 diabetes mellitus and obesity drug development.

the gut is the largest hormone-producing organ of the body. While it has long been known that the presence of nutrients such as glucose, fat, and protein in the intestinal lumen stimulates secretion of many gut hormones, the effects of fructose are poorly understood. While some studies have shown that fructose does not stimulate insulin secretion in: *1*) humans when perfused intraduodenally, *2*) in rat pancreatic preparations, or *3*) in isolated human and rat pancreatic islets (3, 6, 27), other studies showed that fructose causes insulin secretion in humans after oral ingestion (9, 24). Nowadays, fructose is a major sweetener in Western diets (2, 10). However, increased dietary intake of fructose has been suspected to be partly responsible for the growing rates of obesity and the metabolic syndrome (hypertension, hypertriglyceridemia, hyperlipidemia, insulin resistance, and type 2 diabetes mellitus) (17, 20), possibly due to fructose-induced perturbation of cell signaling and inflammatory reactions in insulin-sensitive tissues (21). We conducted the present study to investigate the effect of fructose on appetite- and metabolism-regulating hormones from the gut, with a particular focus on the incretin hormones glucose-dependent insulinotropic polypeptide (GIP) and glucagon-like peptide 1 (GLP-1), since recent expression profiles of some gut endocrine cells suggested expression of the fructose transporter, GLUT5 (14, 19). Because it turned out that fructose clearly stimulated GLP-1 secretion in humans, we investigated intracellular mechanisms behind fructose-stimulated secretion in the murine L cell model, the GLUTag cell line (4), after confirming that GLP-1 secretion was also stimulated in rats and mice.

## MATERIALS AND METHODS

### Effects of Fructose on Gut Hormone Secretion in Humans

#### Subjects and study protocol.

Nine healthy volunteers (4 men and 5 women; mean age 27.7 ± 1.2 yr, range: 23.4–36.8, mean body mass index 21.7 ± 0.4 kg/m^2^, range: 18.2–26.3) participated in the study, which was approved by the local ethical committee and conducted according to the principles of The Helsinki Declaration. All subjects had normal fasting blood glucose levels (5.29 ± 0.39 mM, range: 4.5–6.1 mM), and none had parents or siblings diagnosed with any type of diabetes. No subjects received medication known to interfere with glucose homeostasis. Each subject was studied on two occasions within 3 wk after the first day of study. Subjects were instructed to refrain from vigorous exercise and alcohol for at least 24 h before each study. Study days began at 0830 preceded by a 10-h overnight fast. Venous blood samples were collected at *time −10*, *0*, *15*, *30*, *45*, *60*, *90*, *120 min* in prechilled EDTA (10.8 mg) tubes (catalog no. 367864; BD Biosciences, Albertslund, Denmark) through a polyethylene cannula placed in a cubital vein. At *time 0 min*, the seated, single-blinded, subjects drank a sugar solution containing 75 g fructose or glucose dissolved in 300 ml water (RT) within 2 min. For palatability, solutions were refreshed with lemon juice. Upon collection, blood samples were instantly chilled on ice and centrifuged (2,400 *g*, 15 min, 5°C) within 30 min. Plasma were stored at −20°C until analysis.

### Effects of Fructose and Glucose Gavage in Rats

#### Animals and study protocol.

Studies were approved by the Danish Animal Experiments Inspectorate (2013-15-2934-00833). Male Wistar rats were obtained from Taconic (Ejby, Denmark) and allowed at least 1 wk of acclimatization before the day of experiment. Rats followed a 12:12-h light-dark cycle with ad libitum access to standard chow and drinking water. Every day for a week up to the experiment, rats were handled to minimize stress-related responses on the day of study. Handling included restraint and gavage feeding with drinking water. Experiments were carried out on two occasions on nonfasted rats (294.9 ± 3.8 g) just before their nocturnal feeding period (1700). Weight did not differ between groups or study occasions (*P* > 0.05) (data not shown). Rats were divided into weight-matched groups (*n* = 4/occasion) and given a bolus of either glucose or fructose (2 g/1,000 g body wt) diluted in milli-Q water to a final concentration of 50% (wt/vol) or a matched volume vehicle (milli-Q water). Rats from the same cage received different treatments. Blood (200 μl) was collected into prechilled EDTA-coated capillary tubes (catalog no. 200 K3E, Microvette; Sarstedt, Nümbrecht, Germany) by sublingual vein puncture and instantly transferred onto ice. The zero sample was collected 10 min before bolus administration. At *time 0 min* rats were stimulated with either vehicle, glucose, or fructose, and blood was collected at *time 15*, *30*, and *60 min*. Blood glucose was measured instantly after collection, and samples were centrifuged (1,650 *g*, 4°C, 10 min) to obtain plasma, which was transferred to fresh Eppendorf tubes and immediately frozen on dry ice. Samples were stored at −20°C until analysis. At the end of the experiment rats were anesthetized by subcutaneous injection of hypnorm/midazolam (0.2 ml/100 g, concentration 5 mg/ml) and killed by diaphragm perforation.

### Effects of Fructose and Glucose Gavage in Mice

#### Animals and study protocol.

Studies were carried out with permission from the Danish Animal Experiments Inspectorate (2012-15-2934-00207). Female C57BL/6 mice were obtained from Charles River (Sulzfeld, Germany) and allowed 1 wk of acclimatization before the day of experiment. Mice (16 mice/group, weight: 20.30 ± 0.31 g, 8–10 wk) were fasted overnight but allowed free access to water. Mice were prestimulated with a gavage of 100 μl drinking water at *time −30 min* and again at *time 0 min* with fructose or glucose (3 g/kg) diluted in drinking water to a final concentration of 20% (wt/vol). At *time 0* and *6 min*, blood (75 μl) for hormone measurement was collected into prechilled EDTA-coated capillary tubes (catalog no. 164213; Vitrex, Herlev, Denmark) supplemented with dipeptidyl peptidase 4 inhibitor (2 μl, 1 mM diprotinin A) (catalog no. I9759; Sigma Aldrich). After collection, blood samples were immediately transferred to Eppendorf tubes supplemented with 7.5 μl diprotinin A (1 mM) and centrifuged (3,200 *g*, 20 min, 4°C) within 30 min. Plasma was transferred to fresh Eppendorf tubes, snap-frozen on dry ice, and stored at −20°C until analysis. Blood for glucose measurements (3 μl) was obtained by tail puncture immediately after blood sampling and directly processed for blood glucose measurements as described below. After the first day of study (13 wk), the procedure was repeated. At both occasions, mice were randomly divided into glucose- and fructose-receiving groups. Weights did not differ between fructose and glucose groups at the respective days of study (weight: *week 0*: F = 20.30 ± 0.313 g vs. glucose = 20.59 ± 0.267 g; *week 13*: F = 27.26 ± 1.013 g vs. glucose = 28.49 ± 0.892 g, *P* ≥ 0.37). At the end of the experiment, mice were killed by cervical dislocation.

### GLUTag Cell Studies

#### Cell culture and secretion studies.

GLUTag cells were cultured in T75 cell culture flasks in low-glucose (1.0 g glucose/l) DMEM 5564 medium (Sigma Aldrich, Buchs, Germany) supplemented with 10% (vol/vol) FCS, 1% (vol/vol) penicillin/streptomycin, and 1% (vol/vol) glutamine. Cells were incubated at 37°C, 5% CO_2_ until 70–80% confluent, then trypsinized and plated (×10, 1 ml/well) on a 24-well plate precoated with matrigel (1:100) (catalog no. 354234; BD Biosciences, Bedford, MA) as described previously (15). The following day, cells were thoroughly washed with saline buffer (138 mM NaCl, 4.5 mM KCl, 4.2 mM NaHCO_3_, 1.2 mM NaH_2_PO_4_, 2.5 mM CaCl_2_, 1.2 mM MgCl_2_, and 10 mM HEPES) supplemented with 0.1% (wt/vol) fatty acid-free BSA (A-603-10G; Sigma Aldrich) and incubated for 2 h (37°C, 5% CO_2_) with 250 μl test reagents (fructose, 100 μM gliclazide/tolbutamide, 340 μM diazoxide) dissolved in saline buffer. All reagents were supplied by Sigma Aldrich. Supernatants were collected and centrifuged (1,500 *g*, 5 min, RT) to remove floating cells and debris and then either snap-frozen on dry ice and stored at −80°C or directly processed for GLP-1_active_ measurement.

#### NAD(P)H imaging.

Intracellular NAD(P)H concentration levels were measured using NAD(P)H's autofluorescence properties (22, 23). GLUTag cells were plated on glass bottom dishes (catalog no. P35G-0-14-C; MatTek, Ashland, UK), and experiments were performed using an inverted fluorescence microscope (Olympus IX71) with an ×40 oil-immersion objective as described previously (13, 15). In brief, cells were excited at 360/15 nm using a 75-W xenon arc lamp and monochromator, and emission was recorded at 510/80 nm with an Orca-ER CCD camera (Hamamatsu, UK), controlled by MetaFluor software. Measurements were obtained every 10 s and background corrected using parallel measures from a cell-free location. Cells were perifused with test reagent dissolved in saline buffer (0.1, 1, 3, 10, or 30 mM fructose, 30 mM mannitol, or 10 mM glucose) at a rate of ∼1 ml/min. Glucose was included in all experiments as positive control because of previous studies demonstrating that glucose is metabolized by the GLUTag cell (19). Negative glucose responders (≈1/3) were excluded from the data analysis. Between test agents, cells were washed with saline buffer as above until readings returned to baseline. NAD(P)H imaging data are expressed as background-subtracted average values taken over a period of 5 min before and after test substance application.

#### Biochemical measurements.

GLUTag GLP-1 secretion was measured by an active GLP-1 ELISA kit (catalog no. EGLP-35K; Millipore, Watford, UK) read on a SpectraMax M5 plate reader controlled by SoftMax Pro 5.4.1 software (Molecular Devices, Berkshire, UK). For both human, rat and mouse, blood glucose levels were measured by the glucose oxidase method immediately after sampling by a handheld Accu-chek Compact plus device (Roche, Mannheim, Germany). For the rat, plasma GIP_total_ (GIP_1–42_ and GIP_3–42_) was measured with GIP-ELISA (catalog no. EZRMGIP-55K; Millipore), whereas GLP-1 levels were measured by the same Millipore GLP-1 (active) ELISA kit as for the GLUTag cells. In both cases, data were acquired using a Biotek EL800 plate reader controlled by Gene5 software (BioTek, Brøndby, Denmark). For the mouse, GIP_total_ concentrations were measured as described above, and GLP-1 levels were determined by the Meso Scale assay system [catalog no. L45JVA-1, Custom assay; GLP-1_total_ (version 2), Meso Scale Discovery, Gaithersburg, MD]. Data were obtained with a Sector Imager 2400 device controlled by MSD Discovery workbench software. Human plasma concentrations of total GLP-1, GIP, insulin (INS), glucagon (GCG), and intact neutrotensin_1–13_ (NTS_1–13_) were determined using in-house radioimmunoassays (assay code: GLP-1_total_, 89390; GIP_total_, 80867-5; INS, 2004-3; GCG, 4305-8; NTS_1–13_, 3844-5) with ^125^I-labeled tracers and hormone-specific antiserum, recognizing COOH-terminal epitopes, as described previously (11, 16, 12a, 12b). Free and bound moieties were separated by addition of (bovine) plasma-coated charcoal (E. Merck, Darmstadt, Germany). Supernatants were collected (2,400 *g*, 30 min, 4°C), and the radioactivity was counted in a Packard Cobra II Gamma Counter (GMI). Plasma polypeptide YY_3–36_ (PYY_3–36_) levels were measured by a commercially available RIA kit (catalog no. PYY-67HK; Millipore). Plasma cholecystokinin (CCK) concentrations were measured by RIA measuring all the tyrosyl-sulfated forms of CCK as described (18). For all commercial kits, manufacturers' protocols were followed.

### Data Presentation and Assessment of Statistical Significance

Data are expressed as means + 1 SE. Graphs were constructed using Graphpad Prism 5 software, and statistical significance was calculated with the same program. Data were evaluated statistically by one-way ANOVA followed by Bonferroni post hoc test, Student's *t*-test, paired Student's *t*-test, or one-sample *t*-test, testing values against a hypothetical value of zero, as indicated. *P* < 0.05 was considered significant. For the human study, baseline values are presented as the mean of the −15 and 0 min levels. EC_50_ value for fructose-stimulated GLP-1 secretion from GLUTag cells (see Fig. 5*D*) was obtained by a five-parameter logistic equation using GraphPad Prism software.

## RESULTS

### Fructose Elevates Blood Glucose Levels and Stimulates Insulin Secretion in Healthy Young Humans

#### Glucose.

**Fig. 1.**
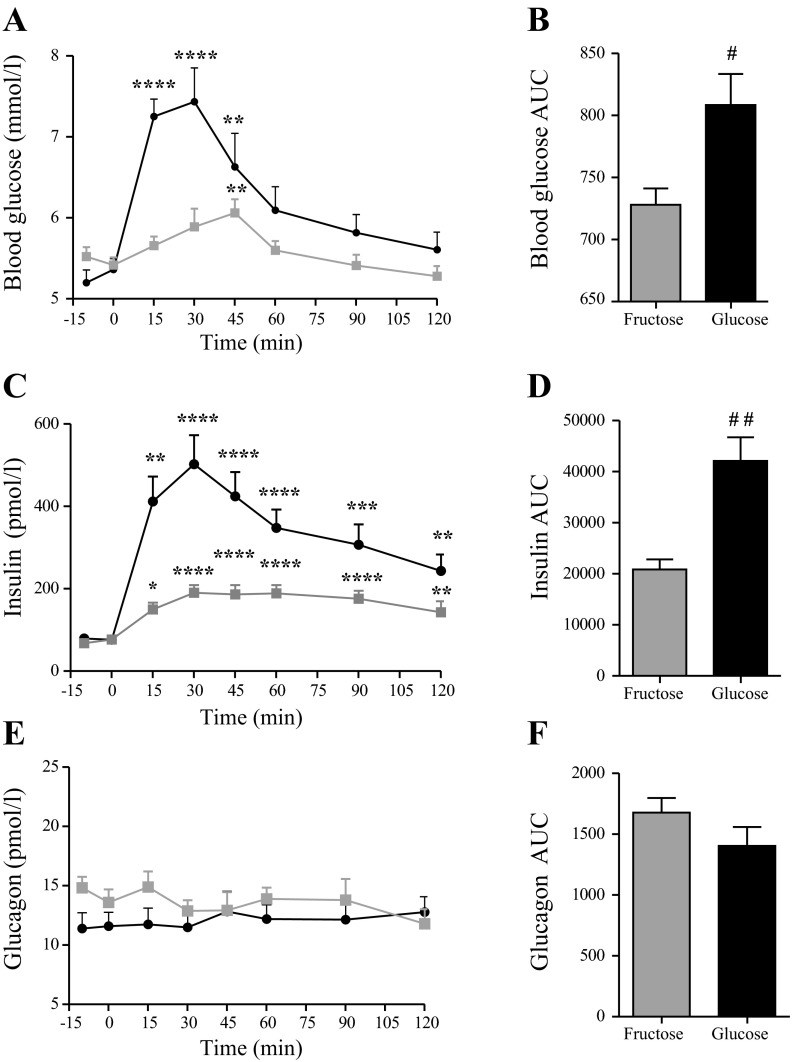
Blood glucose and plasma insulin and glucagon responses to oral glucose and fructose challenge in healthy humans. Mean values + 1 SE are shown in response to oral glucose (black) and fructose (gray) stimulation. Total area under the curve (AUC) values are shown for comparison between treatments. *A*: blood glucose (mM); *B*: blood glucose AUC values (min × mM); *C*: plasma insulin (pM); *D*: plasma insulin AUC values (min × pM); *E*: plasma glucagon (pM); *F*: plasma glucagon AUC values (min × pM). *Within-group significance compared with baseline (mean value for −15 and 0 min control samples). #Level of between-group significance. Within-group significance was tested by one-way ANOVA for repeated measurements followed by Bonferroni post hoc test. Between-group significance was tested by two-tailed paired *t*-test on AUC values. *^/#^*P* < 0.05, **^/##^*P* < 0.01, ***P < 0.001, and ****P < 0.0001, *n* = 9.

There was no difference in blood glucose concentrations at baseline between the glucose and fructose treatment days (*P* > 0.05, *n* = 9) (Fig. 1*A*). Both treatments increased blood glucose (*P* < 0.001) with significantly higher area under the curve (AUC) values after oral glucose vs. fructose (*P* < 0.05, *n* = 9) (Fig. 1*B*). Maximum blood glucose concentrations were also higher in the glucose group (glucose = 7.5 ± 0.4 pM vs. fructose = 6.1 ± 0.2 pM, *P* < 0.05, *n* = 9). For both groups, blood glucose concentrations returned to basal levels by the end of the study period (2 h) (Fig. 1*A*).

#### Insulin.

Fasting insulin concentrations did not differ between groups (*P* > 0.05, *n* = 9). There was a rise in insulin after both glucose and fructose ingestion (*P* < 0.001, *n* = 9), albeit glucose elicited a higher maximal response (glucose = 502 ± 70 pM vs. fructose = 190 ± 19 pM, *P* < 0.01, *n* = 9) (Fig. 1*C*). Insulin AUC was greater after glucose than after fructose (*P* < 0.05) (Fig. 1*D*). Insulin concentrations were still elevated at the end of the study for both treatments (*P* < 0.001) (Fig. 1*C*).

#### Glucagon.

Glucagon levels did not change significantly at any time point after glucose or fructose intake (*P* > 0.05) (Fig. 1*E*). Glucagon AUCs did not differ between treatments (*P* = 0.17) (Fig. 1*F*).

### Fructose Induces CCK, GLP-1, PYY, and NTS but not GIP Secretion in Healthy Young Humans

#### GLP-1_total_.

**Fig. 2.**
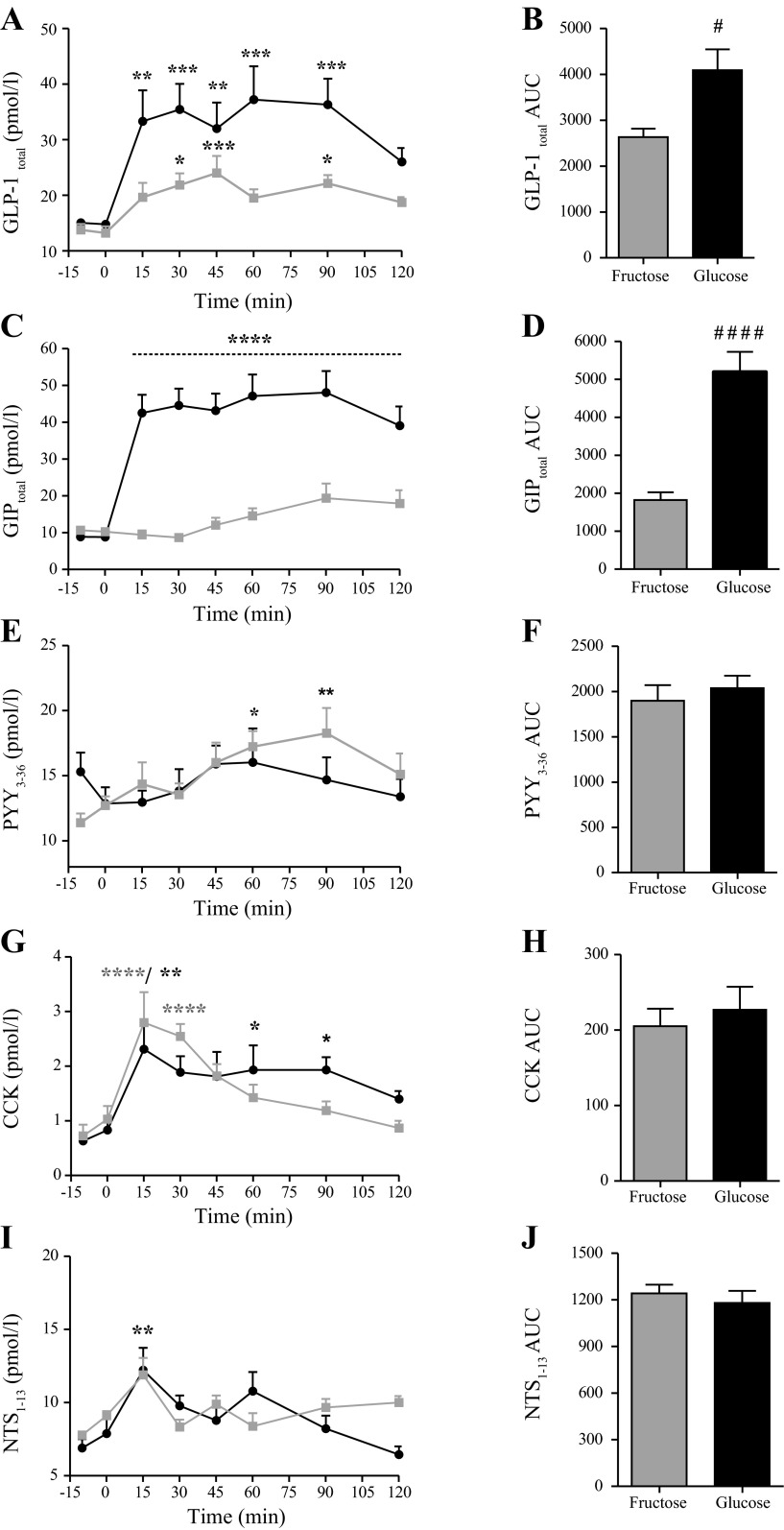
Enteroendocrine hormone responses to oral glucose and fructose challenge in healthy humans. Mean values + 1 SE are shown in response to oral (75 g in 300 ml water) glucose (black) and fructose (gray) intake. Total AUC values are shown for comparison between treatments. *A*: plasma total glucagon-like peptide-1 (GLP-1_total_, pM); *B*: plasma GLP-1_total_ AUC values (min × pM); *C*: plasma total glucose-dependent insulinotropic polypeptide (GIP_total_, pM); *D*: total plasma GIP_total_ AUC values (min × pM); *E*: plasma peptide YY (PYY)_3–36_ values (pM); *F*: Plasma PYY_3–36_ AUC values (min × pM); *G*: plasma cholecystokinin (CCK) values (pM); *H*: plasma CCK AUC values (min × pM); *I*: plasma neutotensin (NTS)_1–13_ values (pM); *J*: plasma NTS_1–13_ AUC values (min × pM). *Within-group significance compared with base level (mean value for −15 and 0 min control samples). ^#^Between-group significance. Significance was tested by one-way ANOVA for repeated measurements followed by Bonferroni post hoc test or paired *t*-test. ^#/^*P < 0.05, **P < 0.01, ***P < 0.001, and ^####/^****P < 0.0001, *n* = 9.

Basal GLP-1 levels did not differ between treatments (*n* = 9) (Fig. 2*A*). GLP-1 concentrations increased significantly after oral fructose intake (*P* < 0.01, *n* = 9) with progressively increasing concentrations from baseline until 45 min (*P* < 0.001). Levels then tended to fall, reaching an elevated plateau phase from 60 to 120 min. GLP-1 also rose after oral glucose intake (*P* < 0.0001, *n* = 9), reaching significantly elevated concentrations at 15 min (*P* < 0.01). Thereafter, levels remained elevated until 90 min (Fig. 2*A*). Over the entire time course, GLP-1 levels were higher after glucose than after fructose stimulation (*P* < 0.05) (Fig. 2*B*).

#### GIP_total_.

GIP concentrations did not differ between treatments at baseline (*P* > 0.05, *n* = 9) (Fig. 2*C*). While GIP concentrations rose significantly at 15 min after glucose ingestion (43 ± 5.0 pM, *P* > 0.0001, *n* = 9), reaching at plateau from 15 to 120 min (*P* < 0.0001, *n* = 9), fructose did not significantly elevate GIP concentrations (*P* > 0.05, *n* = 9), also compared with basal levels (*P* > 0.05) (Fig. 2*C*). GIP AUCs were greater after glucose than fructose intake (*P* < 0.0001) (Fig. 2*D*).

#### PYY_3–36_.

PYY concentrations did not differ between treatments at baseline (*P* > 0.05, *n* = 9) (Fig. 2*E*). PYY concentrations did not reach significantly elevated values following glucose intake (*P* > 0.05, *n* = 9), but levels were slightly elevated at 60 and 90 min after fructose intake (fructose at 0 min = 12 ± 0.5 pM vs. 60 min = 16 ± 1.5 pM and 90 min = 18 ± 1.9 pM, *P* < 0.05, *n* = 9) (Fig. 2*E*). Plasma PYY AUCs did not differ between treatments (AUC values: *P* > 0.05) (Fig. 2*F*).

#### CCK.

CCK concentrations did not differ significantly between treatments at baseline (*P* > 0.05, *n* = 9) (Fig. 2*G*). Glucose and fructose both caused a rise in CCK concentrations (*P* < 0.01, *n* = 9) with peak values occurring 15 min after ingestion for both treatments (glucose = 2.3 ± 0.5 pM vs. fructose = 2.8 ± 0.6 pM, *P* < 0.05, *n* = 9). Thereafter, CCK concentrations decreased for both treatments, albeit with a tendency toward higher values in the glucose treatment group at later time points (Fig. 2*G*). CCK AUC values did not differ between treatments (*P* > 0.05) (Fig. 2*H*).

#### NTS_1–13_.

Basal concentrations were not significantly different between treatments (*P* > 0.05, *n* = 9) (Fig. 2*I*). Glucose and fructose both elevated NTS levels (*P* < 0.001), with similar peak values observed at 15 min for both treatments (glucose = 12 ± 1 pM vs. fructose = 12 ± 2 pM, *P* < 0.01, *n* = 9). NTS AUCs did not differ between treatments (*P* > 0.05) (Fig. 2*J*).

### Fructose Gavage Stimulates GLP-1, but not GIP, in the Rat

**Fig. 3.**
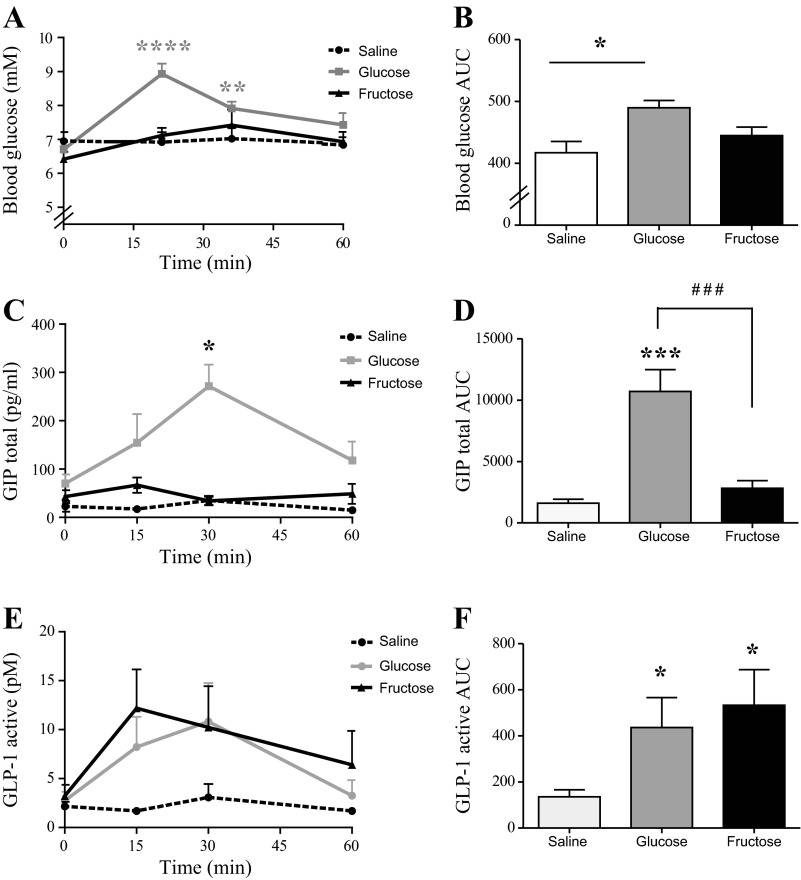
Blood glucose and plasma GLP-1 and GIP responses to oral glucose and fructose stimulation in the rat. Mean values + 1 SE are shown in response to oral glucose (gray), fructose (blue), and saline (black) intake. *A*: blood glucose levels; *B*: blood glucose AUC levels (min × mM); *C*: plasma GIP_total_ levels (pg/ml); *D*: plasma GIP_total_ AUC values (min × pg/ml); *E*: plasma GLP-1_active_ concentrations (pM); *F*: plasma GLP-1_active_ AUC values (min × pM). Between-group significance was tested by two-tailed paired *t*-test on AUC values. *Within-group significance. ^#^Between group significance. **P < 0.01, ^###/^***P < 0.001, *P < 0.05, and ****P < 0.0001 relative to base level, *n* = 7 for glucose and *n* = 6 for fructose and saline.

Oral glucose significantly elevated blood glucose levels at 15 and 30 min (basal level: 6.7 ± 0.1 mM vs. 15 min: 9.0 ± 0.3 mM, 30 min: 7.9 ± 0.2 mM, *P* < 0.01, *n* = 7). At 60 min, blood glucose levels had normalized and were not different from basal levels (*P* > 0.05) (Fig. 3*A*). Blood glucose AUC values were significantly different from saline in the glucose group (*P* < 0.05, *n* = 6) but not in the fructose-treated group (*P* > 0.05, *n* = 6) (Fig. 3*B*). Blood glucose concentrations did not differ at any time points in both control groups (*P* > 0.05, *n* = 6) (Fig. 3*A*). GIP_total_ levels increased after glucose stimulation, reaching significance at 30 min after stimulation (base level: 70 ± 19 pg/ml vs. 30 min: 271 ± 45 pg/ml, *P* < 0.001, *n* = 7). In contrast, fructose stimulation did not elevate GIP_total_ levels at any time points (*P* > 0.05, *n* = 6) (Fig. 3*C*). GIP AUC values were significantly elevated after glucose stimulation compared with control (*P* < 0.001), but not after fructose (Fig. 3*D*). GLP-1 levels were unaltered by water stimulation (*P* > 0.05, *n* = 6), but GLP-1 AUCs were elevated by both glucose and fructose compared with control (*P* < 0.05) (Fig. 3*F*).

### Fructose Gavage Stimulates GLP-1, but not GIP, in Mice

**Fig. 4.**
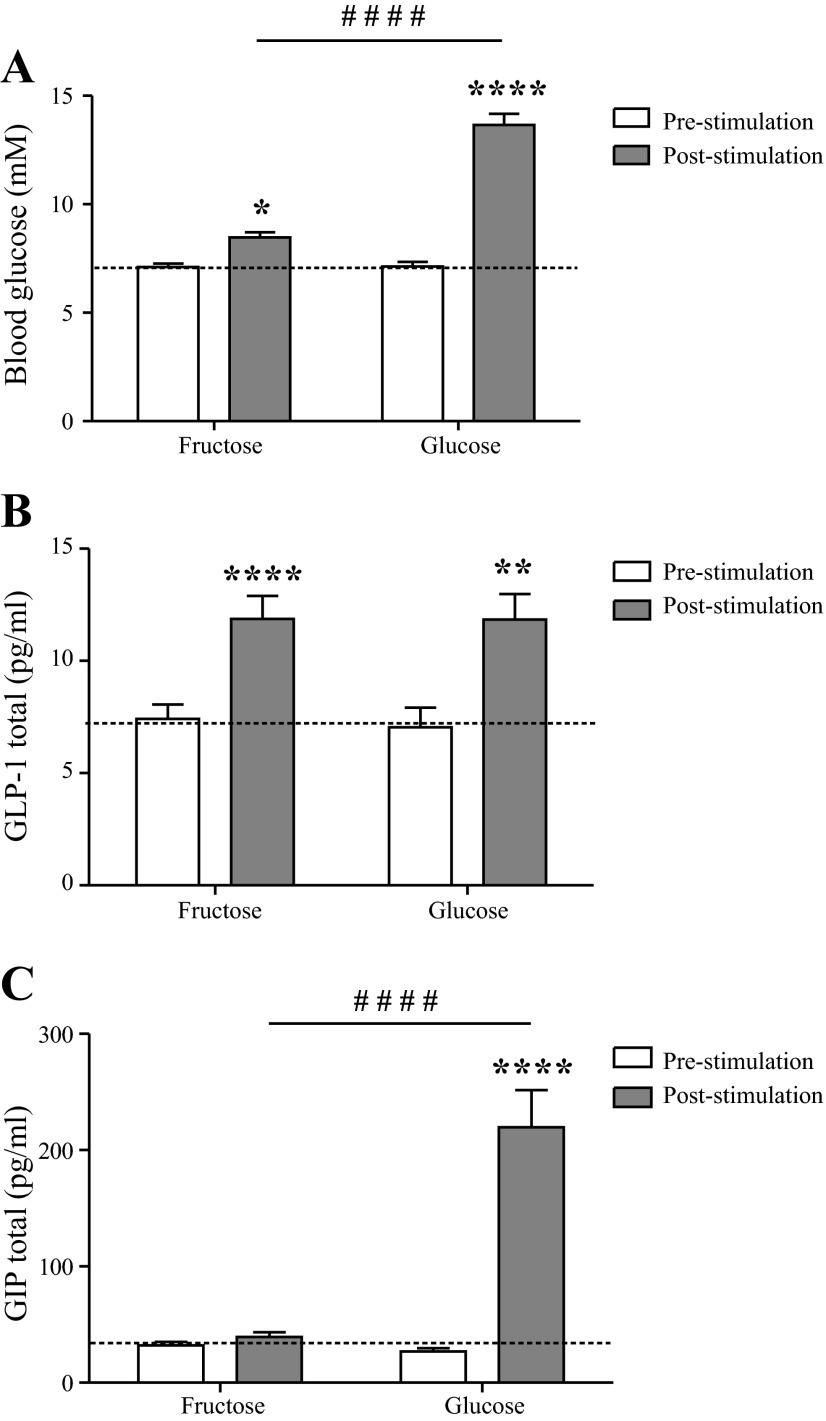
Blood glucose and plasma GLP-1 and GIP responses to oral glucose and fructose stimulation in the mouse. Values are shown as means ± 1 SE at 0 min (white bars, base level) and 6 min (gray bars, response). *A*: blood glucose concentrations (mM); *B*: plasma GLP-1_total_ levels (pg/ml); *C*: plasma GIP_total_ concentrations (pg/ml). Data were analyzed by one-way ANOVA followed by Bonferroni post hoc test or Student's *t*-test. *Within-group significance. ^#^Between-group significance. ****^/####^*P* < 0.0001, ***P < 0.001, **P < 0.01, and *P < 0.05, *n* = 31/group in *A* and *B* and 24–29/group in *C*.

Responses to glucose and fructose were studied on two occasions separated by 13 wk. Blood glucose increased significantly for both groups compared with basal levels (fructose: basal level = 7.1 ± 0.2 mM vs. response = 8.5 ± 0.3 mM, glucose: basal level = 7.1 ± 0.2 mM vs. response = 14 ± 0.5 mM, *P* < 0.05, *n* = 31) albeit glucose stimulation increased levels to a greater extent than fructose (*P* < 0.0001) (Fig. 4*A*). GLP-1_total_ release was stimulated by both treatments (fructose = 7.9 ± 0.8 vs. 12 ± 1.0 pg/ml, glucose: 7.0 ± 0.9 vs. 11.7 ± 1.2 pg/ml, *P* < 0.01, *n* = 23) (Fig. 4*B*) to a similar extent (*P* > 0.05). GIP_total_ concentrations were elevated by glucose treatment (28 ± 2.9 vs. 223 ± 34 pg/ml, *P* < 0.01, *n* = 23), but fructose had no effect (32 ± 3 vs. 40 ± 4 pg/ml, *P* > 0.05, *n* = 23) (Fig. 4*C*). Basal levels of blood glucose, GLP-1, and GIP did not differ between treatment groups (*P* > 0.05) (Fig. 4, *A*–*C*).

### Fructose is Metabolized by the GLUTag Cell and Stimulates GLP-1 Secretion in a Dose-Dependent Manner by ATP-Sensitive Potassium Channel Closure

**Fig. 5.**
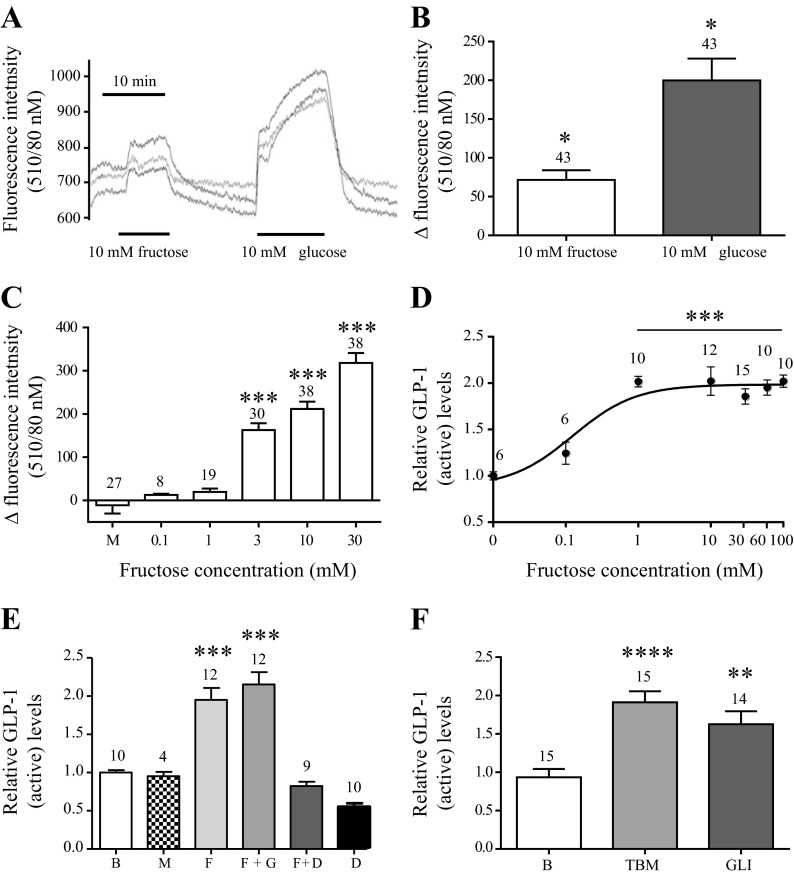
Fructose is metabolized by the GLUTag cell and stimulates GLP-1 secretion dose dependently by ATP-sensitive potassium (K_ATP_) channel closure. Data are presented as mean values ± 1 SE with sample size indicated over the respective bars. Responses are shown relative to base level (B) secretion measured in parallel on the same day. *A*: representative trace of changes in intracellular NAD(P)H levels [fluorescence intensity (340/10 nM)] in response to 10 mM fructose/glucose. Each trace represents one cell. *B*: collected changes in NAD(P)H levels in response to 10 mM fructose and glucose. *C*: changes in NAD(P)H levels in response to depicted concentrations series of fructose (F) and mannitol (M) (osmolarity control). *D*: relative GLP-1_active_ levels in response to concentrations of fructose or as indicated. EC_50_ = 0.155 mM. *E*: relative GLP-1_active_ secretion levels in response to mannitol (100 mM), fructose (10 mM), fructose (10 mM) + gliclazide (10 μM) (F + GLI), fructose (10 mM) + diazoxide (340 μm) (F + D), and diazoxide (340 μM) (D). *F*: relative GLP-1_active_ levels in response to 10 μM tolbutamide (TBM) and gliclazide (GLI). Statistical significance was tested by Student's *t*-test (*B*) or one-way ANOVA analysis followed by Bonferroni post hoc test (*C*–*F*). *P < 0.05, **P < 0.01, ***P < 0.001, and ****P < 0.0001 relative to base level.

Metabolic responses to fructose were investigated by the NAD(P)H imaging technique. Representative traces from an experiment are shown in Fig. 5*A*. Fructose stimulated NAD(P)H production in a dose-dependent manner at a concentration of 3 mM and above, although at 10 mM it was less effective than equimolar (10 mM) glucose (*P* < 0.05, *n* = 43) (Fig. 5*B*). Stimulation with 100 mM of the metabolically inactive sugar mannitol (7) confirmed that the changes were not related to osmolar stress (*P* > 0.05, *n* = 27) (Fig. 5*C*). Fructose stimulated GLP-1 release from the GLUTag cell line (*P* < 0.0001) in a dose-dependent manner, reaching a maximal response at 1 mM (EC_50_ = 0.155 mM, *P* < 0.001, *n* = 6–16) (Fig. 5*D*). Again, this was not related to osmolar stress, since 100 mM mannitol did not increase GLP-1 release (*P* > 0.05, *n* = 4–12). Addition of gliclazide (GLI) to induce ATP-sensitive potassium (K_ATP_) channel closure did not potentiate fructose-induced GLP-1 secretion (*P* > 0.05, *n* = 12), but stimulation with GLI alone caused GLP-1 secretion to a similar degree as tolbutamide (*P* < 0.01, *n* = 14–15) (Fig. 5*F*). Presence of the K_ATP_ channel opener diazoxide (340 μM) completely abolished fructose-induced GLP-1 secretion (fructose = 2.0 ± 0.2 vs. fructose + diazoxide = 0.8 ± 0.1, *P* < 0.001, *n* = 10), which reached similar levels as during diazoxide exposure alone (fructose + diazoxide = 0.8 ± 0.1 vs. diazoxide = 0.6 ± 0.1, *P* > 0.05, *n* = 10–12) (Fig. 5*E*).

## DISCUSSION

The increased intake of dietary fructose in general and high-fructose corn syrup in particular has been suggested to play a role in the current obesity epidemic (8, 20). Moreover, fructose has been suggested to have a lower satiating potency than glucose, but the available studies are conflicting and involve fructose doses greater than normally consumed, as reviewed (12). In this study, we examined the effects of fructose intake on the secretion of gut hormones, known to influence appetite and metabolism. The main finding was that fructose intake caused GLP-1 secretion in healthy humans, whereas it had no effect on GIP secretion. Fructose also stimulated the release of CCK and NTS_1–13_ to a similar extent as glucose, and also enhanced insulin secretion, albeit to a lower degree than glucose. In contrast, neither fructose nor glucose altered glucagon or PYY_3–36_ levels. With a view to perform mechanistic studies regarding the differential responses of the incretin hormones, we also investigated GIP and GLP-1 responses to fructose in mice and rats. In both animals, fructose stimulated GLP-1 secretion to the same extent as isocaloric glucose, but, similar to humans, GIP secretion was not stimulated.

In mice and humans, fructose intake also increased blood glucose levels, and a similar tendency was seen in the rat. It is generally accepted that fructose can be converted to glucose in the liver by mechanisms involving rapid phosphorylation of fructose to fructose 1-phosphate by fructose kinase, which by liver-type-B aldolase activity, yields the intermediate metabolites dihydroxyacetone phosphate and glyceraldehyde, with the latter being a substrate for glucose production by further metabolic activity. However, it has also been shown that the small intestine has capacity to transform fructose into glucose by the same pathway (1, 5). Although not investigated here, it may be speculated that the rise in blood glucose after fructose intake reflects a combination of intestinal and hepatic conversion. Previous studies have shown that fructose did not stimulate insulin secretion from perfused rat pancreatic preparations or from isolated human and rat pancreatic islets (3, 6, 27), but two other human studies found, in agreement with our study, that oral fructose stimulates insulin release and showed that fructose is a less effective insulin secretagogue than glucose (9, 24). This would suggest that the stimulatory effect in humans might result from the effects of the increased glucose concentrations in combination with the secretion of the insulinotropic hormone, GLP-1. GIP clearly did not contribute.

Because fructose stimulated GLP-1 release also in the mouse, this provided a meaningful reason to study the underlying mechanism of GLP-1 release using the murine L cell model, the GLUTag cell line. A direct mechanism might be expected since expression of the fructose transporter GLUT5 was previously found in both L cells and GLUTag cells. We reported previously that glucose metabolism stimulates GLP-1 secretion from GLUTag cells by K_ATP_ channel closure, causing cell membrane depolarization (13), consistent with high expression levels of K_ATP_ channel subunits Kir6.2 and SUR1 in both GLUTag cells and primary murine L cells (19). We show here that fructose was also metabolized by GLUTag cells and that fructose-triggered GLP-1 release was similar in magnitude to that induced by the K_ATP_ channel inhibitors gliclazide and tolbutamide. That fructose did not trigger further secretion when added in the presence of gliclazide suggests that it acts at least in part via K_ATP_ channel closure. This is further supported by the finding that the channel opener diazoxide lowered GLP-1 secretion below basal levels even in the presence of fructose, although it should be noted that the membrane hyperpolarization induced by diazoxide would also inhibit secretion stimulated by other stimuli dependent on membrane depolarization. We cannot, therefore, exclude the possibility that fructose, like glucose, also enhances GLP-1 release from GLUTag cells via a metabolic action independent of K_ATP_ channel closure. Whether fructose also targets similar pathways in primary L cells remains to be established. Our study provides no information on the cellular and physiological basis for why fructose stimulates GLP-1 and CCK, but not GIP, secretion. We reported previously that GLP-1 secretion from primary intestinal cultures was readily triggered by glucose (19), whereas glucose-triggered GIP responses were small in the absence of additional agents that increased cAMP concentrations (14). Thus, although primary murine K cells do express GLUT5 and the K_ATP_ channel subunits (14), one possible explanation for the discrepancy between the cells reported here, therefore, is that K cells are generally less excitable than L cells and that the weaker fructose stimulus is sufficient to activate L cells, but not K cells. It is also possible, however, that the more distal location of L cells renders them more responsive to fructose. The sugar may, for example, be fermented by the gut microbiome, producing local stimuli such as short-chain fatty acids (25), although this is unlikely to explain the acute effects of fructose seen 15 min after ingestion.

The metabolic consequences of the enhancement of GLP-1 but not GIP secretion may be important, since it appears to be the first time that a nutrient has been shown to activate the L cells while not affecting the K cells in vivo. Fructose might thus be used as a tool to discriminate between the combined actions caused by secretion of both GIP and GLP-1 and the effects caused by GLP-1 alone. Furthermore, because fructose elicits GLP-1 secretion without simultaneous release of glucagonotropic GIP, the pathways underlying fructose-stimulated GLP-1 release might be useful targets for drug development aiming at stimulating GLP-1 secretion for the treatment of type 2 diabetes and obesity. For obesity treatment, this strategy may prove to be a more an effective strategy to reduce food intake than monohormone treatment, since the stimulation of GLP-1 secretion should lead to simultaneous release of the costored anorectic hormones CCK, glicentin, oxyntomodulin, and PYY, as reviewed (26).

## GRANTS

The study was supported by a Wellcome Trust grant to F. M. Gribble (WT088357) and a grant to J. J Holst from the Novo Nordisk Centre for Basic Metabolic Metabolism (Novo Nordisk Foundation, Denmark).

## DISCLOSURES

The authors declare no financial or other conflicts of interest.

## AUTHOR CONTRIBUTIONS

Author contributions: R.E.K., F.M.G., B.H., F.R., J.A.W., and J.J.H. conception and design of research; R.E.K., B.H., J.A.W., and J.F.R. performed experiments; R.E.K., B.H., and J.F.R. analyzed data; R.E.K., F.M.G., B.H., F.R., and J.J.H. interpreted results of experiments; R.E.K. prepared figures; R.E.K. drafted manuscript; R.E.K., F.M.G., B.H., F.R., J.A.W., and J.J.H. edited and revised manuscript; R.E.K., F.M.G., B.H., J.A.W., J.F.R., and J.J.H. approved final version of manuscript.
